# Optimization of short tandem repeats (STR) typing method and allele frequency of 8 STR markers in referring to forensic medicine of Semnan Province

**Published:** 2015

**Authors:** M Eskandarion, M Najafi, M Akbari Eidgahi, A Alipour Tabrizi, T Golmohamadi

**Affiliations:** * Biochemistry Department, Tehran University of Medical Sciences, Tehran, Iran; ** Cellular and Molecular Research Center, Biochemistry Department, Iran University of Medical Sciences, Tehran, Iran; *** Semnan Biotechnology Research Center, Semnan University of Medical Sciences, Semnan, Iran; **** Iranin Legal Medicine Research Center, Legal Medicine Organization, Tehran, Iran

**Keywords:** STR, multiplex PCR, capillary electrophoresis, identification

## Abstract

**Background and Objective:** Short Tandem Repeats (STR) show considerable differences among individuals in the population from which they used for identification. There are various methods for analysis of these STR loci, and capillary electrophoresis method already used as an international standard. Due to the high costs of this process, this study aimed to set up a Multiplex PCR method in some standard STR loci so that we can use its PCR product in STR analysis with different methods of HPLC, GC-Mass, and Capillary Electrophoresis.

**Materials and Methods:** 8 typical STR loci in the identification selected according to their size in the two groups of four (CSF1PO, VWA, D18S51, PentaD and TPOX, Amelogenin, FGA, SE33) from NIST (National Institute of Standards and Technology). The above SSR primers prepared from Genbank and Monoplex PCR was designed based on their size. Then, with the changes in temperature conditions, magnesium ion, primers concentration, and setting-up, Hot Start Multiplex PCR of four markers was carried out. PCR product investigated on the agarose gel electrophoresis (3%) and the results of genotyping analyzed by Genetic Analyzer.

**Results:** The Results showed that all STR loci under study are detectable as Monoplex PCR at a temperature of 62°-66° and 1.5 mM magnesium ion. Moreover, Multiplex PCR results showed that when the concentration of primer and temperature measured by the fixed concentration of magnesium, CSF1PO, and D18S51 loci bands are weaker than desired. Using a standard buffer and set Magnesium conditions against changes in the primer concentration and temperature, when Taq polymerase enzyme is added to test tubes at a temperature of 94°, Multiplex PCR bands are visible desirably. Capillary electrophoresis genotyping results obtained in all eight loci and the Locus FGA had the most allelic diversity and the loci TPOX and CSF1PO had the lowest allelic diversity. TPOX and CSF1PO loci had the lowest allelic frequencies, and FGA locus had the most allelic frequency. Moreover, about the determination of statistical indicators of identification using PowerStats V12 software, CSF1PO locus allocated the most RMP (0.219) and FGA locus the highest heterozygosity (100%) and the highest polymorphic rate (PIC) (0.82).

**Conclusion:** The setup performed in this study showed that with two-step multiplex PCR procedure of four markers, PCR can be carried out for eight loci without additional real-time products that this shows proper conditions that we can use their PCR product in analyzing SRTs with different methods of HPLC, GC-Mass, and Capillary Electrophoresis. Besides, the FGA locus was raised as the best loci for identification in the study population concerning the high PD index and high polymorphism.

## Introduction

The content similarity of genetic material among human beings on the planet is very much despite their apparent differences. Therefore, only a small percentage (about 0.3%) of humans’ DNAs is different. The use of this slight difference that is the criterion for uniqueness of every person makes possible identifying him from other people [**1**]. Polymerase chain reaction procedure used in PCR of small tandem repeats (PCR-STR) as a selection approach in forensic medicine and paternity test as well as in determining the genetic map, demographic studies, class analysis, evolutionary studies, National DNA database acquisition and identification of strains in tissue culture [**1**]. Microsatellites or STR, are loci with alleles of small sequences of 2 to 7 bp [**1**]. These courses are scattered throughout the human genome and show considerable differences among human societies [**2**]. Autosomal STRs used as a useful instrument in identifying persons and determining the parent-child relationship in court and criminal investigations [**2**,**3**]. Another feature of STR markers is that the number of repeats is highly variable among people (even among close relatives). And this specificity enables them in the diagnosis of human identity and distinguishing two individuals [**4**].

These sequences are highly polymorphic and can easily be amplified by PCR method [**4**]. The small size of the fragments (200bp) provides the possibility of examining them from slight amounts of DNA and the degraded sample. Thus, we can explore these areas as a sensitive and accurate selection method in the identification of individuals [**5**]. The FBI organization in the US has used 13 STR areas in a standard manner and has established an extensive database to store DNA profile from which is employed in criminal investigations [**4**].

**The studied loci in this research include**

CSF1PO: This locus is a plain sequence repeat located in Intron 6 of human c-fos proto-oncogene in the long arm of Chromosome 5 and includes AGAT repeat units via alleles between 5 to 17 sizes.

TPOX: is a simple tetra-nucleotide located in intron 10 of TPO gene on the short arm of Chromosome 

2. It has AATG repeat units with the lowest Polymorphism among the 13 FBI loci between 5 to 17 sizes.

VWA is a complex tetra-nucleotide located in intron 40 in von Willebrand Factor gene and on the small Chromosome arm twelve. It named as VWF in some manuscripts. It includes repeating unit sequences (TCTA or TGTG) with 10 to 25 repeats.

SE33: This locus is a marker that has recently been raised by German Core loci (GCL) and does not include within 13 standard set of 13 STR loci. This locus is on Chromosome 6, with the unit repeat of AAAG, which happened for 3 to 49 times.

D18S51 is a pure tetra-nucleotide is on the long arm of Chromosome 18 and includes repeated AGAA sequence ranging in size from 5.3-40 repeats and more than 70 alleles reported from this locus.

PentaD is a pentanucleotide on Chromosome 21 ranging from 1.1 to 19 repeats. However, it may be eliminated in some smaller alleles.

FGA is a combination of tetra-nucleotide repeats realized in the 3rd intron of the person alpha fibrinogen locus on the long arm of Chromosome four with repeated CTTT sequence and the allelic replication of 12-51. Amelogenin: is used to determine gender. Amelogenin gene codes some proteins in the enamel and the result of amplification of this gene for samples with male origin is two different products for Chromosomes X and Y and for samples with female origin is only a product for the X chromosome [**6**]. Since there are various methods for analysis of STR loci, capillary electrophoresis method is currently used as an international standard. Due to the high costs of this process, this study aims at setting up a Multiplex PCR method in some standard STR loci so that we can use the PCR product in STRs analysis with different methods of HPLC, GC-Mass, Capillary Electrophoresis. Also, STR Loci allele frequency is being calculated in referring individuals throughout the world. And in our country, by Paragraph 5 of Article 156 of the Constitution of the Iran, an important duty of the Judiciary (Judicial System) is to try and take appropriate action to prevent crime. Therefore, the allelic frequency determination, heterozygosity, and efficacy of these markers seem necessary to verify identity.

## Materials and Methods

The study population: In a randomized study, venous blood samples were taken from 20 unrelated patients for entry into the study. The purpose of this study and how to cooperate described by the researcher and written consent forms received from the interested persons.

**Sample collection and DNA and PCR –STR extraction**

3 ml of blood was kept in sterile tubes containing EDTA for taking next steps in the Laboratory of Forensic Medicine Center of Semnan in -20°C. DNA extraction was carried out using PrimePrepTM Genomic DNA Isolation Kit of GeNet Bio company. And the concentration of DNA extracted sample determined by a UV spectrophotometer (Ratio of A260/ A280, which is the estimate of DNA purity is 1.7-2). Eight STR loci standard in identification selected in two groups of four: (CSF1PO, VWA, D18S51, PentaD and TPOX, Amelogenin, FGA, SE33) based on allelic frequency size, resolution and greater and lower differentiation. Primers were designed using the NCBI site and blasting in the Gene Bank (**Table 1**).

**Table 1:** STR loci name and some features of the STR loci under examination in this research

Locus	Genbank accession	Allele range	PCR fragment (bp)	Primer sequence (5–3)	TM
*CSF1PO*	X14720	6-15	75–111	F:ACTGCCTTCATAGATAGAAGAT	56.6
				R: GCCCTGTTCTAAGTACTTCCT	59.4
*TPOX*	M68651	6-16	61–101	F: CTTAGGGAACCCTCACTG	64.89
				R: GCAGCGTTTATTTGCCCAA	65.99
*FGA*	M64982	16-51/2	151–293	F:CTCACAGATTAAACTGTAACCA	58.20
				R: TTGTCTGTAATTGCCAGC	57.96
*VWA*	M25858	11-24	121–173	F: TCAGTATGTGACTTGGATTGA	55.5
				R:GTAGGTTAGATAGAGATAGGACAGA	62.5
*D18S51*	X91254	7-27	213–293	F: GTCTCAGCTACTTGCAGG	56.11
				R: GGAGATGTCTTACAATAACAGTTG	60.51
*SE33*	V00481	10-34	450–523	F: AATCTGGGCGACAAGAGTGA	58.4
				R: ACATCTCCCCTACCGCTATA	58.4
*PentaD*	AP001752	30-45	400–429	F: GAAGGTCGAAGCTGAAGT	57.33
				R:ATTAGAATTCTTTAATCTGGACACAAG	60.75
*Amelogenin*	M55418	X,Y	121,127	F: CCCTGGGCTCTGTAAAGA	56.1
	M55419			R: AGGCTTGAGGCCAACCAT	56.1

PCR was performed using thermal cycler (BioRad-Germany) in DNase and RNase-free vials of 0.2 ml. PCR reactions performed in several stages. To verify the function, the received primers were first entered in Monoplex PCR reactions and evaluated regarding product development. To perform the Monoplex PCR test, PCR vial containing 50 ng of DNA from an individual, PCR 1X buffer, and 1.5Mm of MgCl2 (for FGA from MgCl22Mm) used. Besides, 0.6 mM of each of the primers and 200 mM of dNTP (From Taz-Germany) and a Taq polymerase unit (Native Fermentas, Germany) were used. Eight loci of this study classified into two groups of four: CSF1PO, VWA, D18S51, PentaD and TPOX, Amelogenin, FGA, SE33 with the changes in temperature conditions, magnesium ion, primers concentration, and setting-up Hot Start Multiplex PCR of four markers. The schedule was as follows: 

The initial denaturation temperature (95°C up to 3 min), 35 periods of denaturation (94°C up to 30 sec), annealing (64°C for 45 sec), extension (72°C up to 45 sec), and the ultimate extension step phase (72°C for 10 min). 

**Electrophoresis**

PCR product on agarose gel electrophoresis (3%), products size, and the results of Genotypes determined by the genetic analyzer.

**Statistical Analysis**

Allelic frequency distribution, the percentage of heterozygosity and some useful population parameters in paternity and Forensics such as matching probability (PM), discrimination (PD), the exclusion power (EP) and fatherhood state (PS) determined by PowerStats V1.2 software.

**Findings**

To examine the functionality of primers, in the step of primers design, a set of factors such as the melting point, four pairs of primers associated with a QuadruPlex, the size range provided by the alleles after PCR and electrophoresis. The effectiveness of banning four pairs of primers associated with the same QuadruPlex evaluated. Due to these properties, two QuadruPlex systems consisting of eight STR markers: (CSF1PO, VWA, D18S51, PentaD and TPOX, Amelogenin, FGA, SE33) were designed. Then, the tags in a QuadruPlex system were set up. In the following, according to the protocols obtained from Monoplex Systems, a contract was presented for the relevant QuadruPlex protocol systems, and it finally brought to the optimization step with many changes.

**Interpretation of Multiplex systems**

The results showed that at a temperature of 62°-66° and 1.5 mM magnesium ion concentration, all STR loci studied are detectable as Monoplex PCR. Besides, Multiplex PCR primers results also showed that when the concentration of primers and temperature measured at a fixed concentration of magnesium, CSF1PO, and D18S51 loci bands are weaker than desired.

**Fig. 1:**
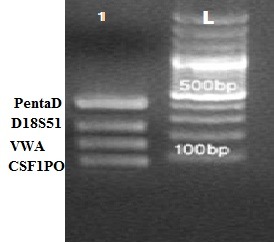
QuadruPlex image (1) taken from the sample under study. This picture has a size marker (Fermentas) with product number 0321 SM on the right side of the gel, and the band size specified at the top of each. Bands are in the expected size range. This gel is Agarose (2.5%)

**Fig. 2:**
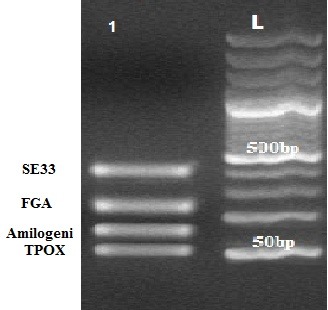
QuadruPlex Image (2) in a sample under study

Using a standard buffer and fixed Magnesium conditions against primer concentration and temperature changes, when Taq polymerase added to the test tubes at a temperature of 94°C, Multiplex PCR bands are visible desirably (**Fig. 1**,**2**).

This image has a size marker (Fermentas) with product number 0371 SM at the right side of the gel that the scale of the bands identified at the top of each. Bands are within the expected size range. The mentioned gel is agarose (2.5%).

**Fig. 3:**
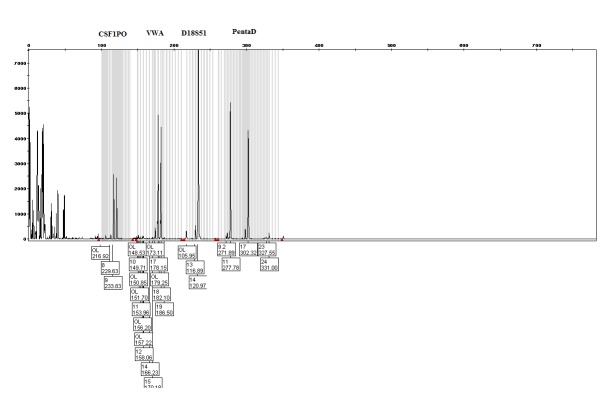
Genetic profile image, shows one subject under study in STR loci (CSF1PO, vWA, D18S51, PentaD) with CE method. Standard size or liz2500 and kit Ladder and Polymer (POP-4) capillary y36 column

Capillary electrophoresis method used for genotyping of STR loci. Using Genetic Analyzer ABI 3130, capillary electrophoresis was carried out. In the end, chromatogram analysis was performed using the Gene Mapper software (**Fig. 3**).

In this study, most polymorphism is observed in locus FGA and then in loci D18S51 and SE33, and the least found in the loci TPOX and CSF1PO (**Table 2**). Most allelic frequency found in locus FGA in Alleles 19, 20, 24 with 18.42% and locus D18S51. Allele 12 with 33.33% and SE33 Allele 40 with 32.5% allocate most allele frequencies to them. In CSF1PO allele 9 and TPOX allele 10, the least rates are observed (**Table 2**).

**Table 2:** Allele Frequency Percent of 7 STR loci in the population (N= 20) in Semnan, Iran

Allele \ Locus	*CSF1PO*	*VWA*	*D18S51*	*PentaD*	*TPOX*	*FGA*	*SE33*
8					44.73		
9	2.63				13.15		
10	15.78				2.63		
11	47.36				36.84		
12	23.68		21.05		2.63		
13	10.53		36.85				
14		7.89	13.16	30			
15		15.78	7.90				
16		18.42	5.26	40			
17		26.31	10.25				
18		28.94		20			
19		2.63				18.42	
20						18.42	
21						10.52	
22						15.79	
23						13.16	
24						18.42	
25						2.63	
27						2.63	
28				7.5			
30				2.5			
36							10
38							35
39							2.5
40							32.5
41							2.5
42							7.5
43							10
All	100	100	100	100	100	100	100

Assessment of STR markers in identification was carried out using allele frequencies obtained in the present study using Software V1.2 Powers tats for the target population. And locus CSF1PO allocated the highest RMP (0.219), and locus FGA assigned the highest heterozygosity (100%) and the highest polymorphic value (PIC (0.82) to themselves (**Table 3**).

**Table 3:** Forensic efficiency parameters Percent of 7 STR loci in the population (N= 20) in Semnan, Iran

The statistical parameters \ Locus	*CSF1PO*	*VWA*	*D18S51*	*PentaD*	*TPOX*	*FGA*	*SE33*
		Forensic Statistics					
RMP	0.219	0.125	0.102	0.175	0.108	0.250	0.260
PD	0.78	0.875	0.898	0.825	0.892	0.750	0.740
PIC	0.65	0.75	0.75	0.60	0.82	0.68	0.68
		Paternity Statistics					
PE	0.679	0.580	0.331	0.212	1.000	0.510	1.000
TPI	3.17	2.38	1.36	1.60	#DIV/	2.00	#DIV/
		Allele Frequencies					
HO	15.8%	21.1%	36.8%	47.4%	0	25%	0
HE	84.2%	78.9%	63.2%	52.6%	100 %	75%	100.0%
P value	P ≤ 0.05	P ≤ 0.05	P ≤ 0.05	P ≤ 0.05	P ≤ 0.05	P ≤ 0.05	P ≤ 0.05
Total Alleles	38	38	38	38	38	38	38

## Discussion and Conclusion

In general, the outcomes of this study can be summarized in two parts:

1- Creating a molecular system for investigating STR loci and optimizing multiplex PCR method to analyze STR loci paves the way for forensic investigations and further studies of these loci in different populations. The set up performed in this study showed that we can perform PCR from 8 loci using two Multiplex PCR reactions of four markers, which this shows proper conditions that we can use their PCR product in STR analyses with different methods of HPLC, GC-Mass, Capillary Electrophoresis.

2. In investigating population studies of allelic diversity, the allelic frequency, heterozygosity and other obtained legal parameters were calculated using CE. FGA locus had the greatest allelic diversity and loci TPOX and CSF1PO had the least allelic diversity. The estimated allelic frequency showed that the loci TPOX and CSF1PO had the least FGA rate, and locus FGA had the greatest Allelic frequency. After comparing with several other performed studies, similar results were observed with some minor differences in allele spectrum, frequency, and the degree of heterozygosity. Locus TPOX has the least polymorphism and heterozygosity in most previous studies [**7**-**12**]. It should also say about allele frequency that the results of this research are different with other research results. Lim et al. in 2005 observed the most allele frequency in the loci CSF1PO, FGA, TPOX, and D18S51 in alleles 12, 23, 8 and 14. Moreover, Muro et al. observed allele frequency in these four loci, in alleles 10, 22, 17 and 11 respectively [**13**,**14**]. In this study, the most allelic frequency was observed in loci CSF1PO, VWA, D18S51, PentaD in alleles 16, 13, 11 and 11, respectively and loci TPOX, FGA, SE33, in Alleles 38, 24 and eight respectively. This difference is due to allele frequencies distribution in different populations. Accordingly, migrations and community roots can be examined. After calculating the legal parameters results of CE method using PowerStats V12 software, RMP value in locus CSF1PO (0.219) allocated the highest value in loci under study (0.219). This indicates that the probability of having a standard profile in the locus CSF1PO for two individuals randomly selected from the population is more than in other loci. Moreover, the property of differentiation (PD) and other indicators of this locus is less than other loci. In contrast, Loci VWA, D18S51, and FGA have the lowest RMP and the most PD. This means that it is less likely to find a standard profile in the population in these loci. In this regard, In Jahromi et al. research among prisoners of Shiraz, FGA and D18S51 had the lowest RMP and the most PD respectively which is consistent with the findings of the current research. This proves the importance of these two loci in identification affairs [**7**]. The highest heterozygosity observed in locus FGA (100%), and lowest heterozygosity found in locus TPOX. It has been found in many studies conducted in Iran and abroad, this locus has accounted for the highest rate of heterozygosity [**7**,**8**,**10**,**15**]. With the evaluation of heterozygosity and other legal parameters, we find that there is a correlation between the heterozygosity level and other parameters related to the identification so that the loci with more heterozygosity have lower RMP and vice versa. According to the results obtained in the population under study, locus FGA had the highest polymorphic value in both methods which was consistent with Jahromi et al results on Prisoners in Fars province [**7**]. Moreover, in studies on Iranians living outside the country, FGA was introduced as the most polymorphic value that the high PD and PIC in Locus FGA offers it as the best locus in identification.

**Acknowledgement**

This article is part of the work of Mohammadreza Iskandar on fulfilling the requirement for a Master of Science. This practice was financially supported by Tehran University of Medical Sciences, in Tehran, Iran.
